# Comparisons of different approaches and incisions of thyroid surgery and selection strategy

**DOI:** 10.3389/fendo.2023.1166820

**Published:** 2023-07-17

**Authors:** Qiyu Lu, Xuemei Zhu, Peisong Wang, Shuai Xue, Guang Chen

**Affiliations:** Department of Thyroid Surgery, General Surgery Center, The First Hospital of Jilin University, Changchun, China

**Keywords:** thyroid surgery, cosmetic outcome, cervical approach, remote-access approach, selection strategy

## Abstract

To date, the traditional open thyroid surgery *via* a low collar incision remains the standard approach for patients undergoing thyroidectomy. However, this conventional approach will inevitably leave patients a neck scar and even cause a variety of complications such as paresthesia, hypesthesia, and other uncomfortable sensations. With the progress in surgical techniques, especially in endoscopic surgery, and the increasing desire for cosmetic and functional outcomes, various new approaches for thyroidectomy have been developed to avoid or decrease side effects. Some of these alternative approaches have obvious advantages compared with traditional surgery and have already been widely used in the treatment of thyroid disease, but each has its limitations. This review aims to evaluate and compare the different approaches to thyroidectomy to help surgeons make the proper treatment strategy for different individuals.

## 1 Introduction

The incidence of thyroid carcinoma has increased rapidly during the past decades worldwide. Owing to the increasing use of ultrasonography, a large number of early tumors have been detected, and most of them have a great prognosis after surgery. As a result, more attention has been paid to increase the patients’ quality of life after surgery. Scarring and neck discomfort after conventional thyroidectomy are two main factors that decrease the patient’s quality of life. In the past two decades, many new approaches have been developed such as the lateral approach (LA), minimally invasive video-assisted thyroidectomy (MIVAT), and transaxillary approach (TA). Some of these alternative approaches have obvious advantages compared with traditional surgery and have already been widely used. However, these new techniques also bring new problems and controversies since each of them has limitations, and it is important to select an ideal approach for patients. In this review, we discuss the different main approaches to thyroid surgery and the selection strategy to help surgeons make the proper treatment strategy for different individuals.

## 2 Cervical approach

### 2.1 Conventional open approach

**Figure 1:**
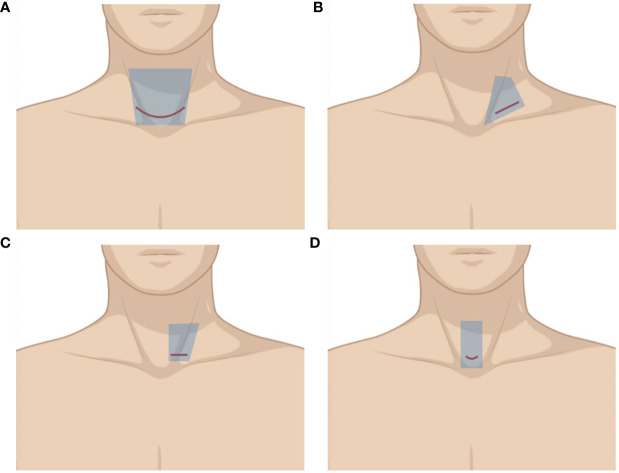
Illustration of different cervical approaches. The area of raised skin flap was marked with blue wireframes. **(A)** Conventional open approach (COA). **(B)** Minimally invasive video-assisted thyroidectomy (MIVAT). **(C, D)** Lateral approach (LA).

Thyroidectomy *via* the conventional open approach (COA), which is firstly proposed by Theodore Kocher in the late 1800s (1), is still the main option for most patients. A collar incision is made along the natural skin fold about one finger breadth above the sternal notch (**Figure 1A**). It gives surgeons great surgical view and operative space compared with other approaches and incisions, which makes the operation easier. For patients with a huge tumor, severe neoplasm invasiveness, or cervical surgery history, COA through a low collar incision might be the first and only choice to guarantee tumor resection and oncological effectiveness. However, it normally requires a long incision, wide skin flaps on the anterior neck, and a long midline opening of the strap muscles to achieve good thyroid exposure. As a result, most patients will have a neck scar more or less, and many of them have uncomfortable sensations such as paresthesia, hypesthesia, and swallowing dysfunction, which bother them a lot after surgery. Sometimes, although not as common as it used to be, an L-shaped or U-shaped incision has to be made on patients with extensive lymphatic metastasis, which can seriously impact their quality of life due to the horrible scar it leaves.

### 2.2 Lateral approach

According to the 2015 American Thyroid Association guidelines (2), thyroid lobectomy might be sufficient for carefully selected patients with 1- to 4-cm well-differentiated thyroid carcinoma. Recently, some surgeons try to modify COA by using a lateral approach. A supraclavicular oblique incision approximately 3–4 cm is made between the anterior edge of the sternocleidomastoid (SCM) and the external jugular vein along the dermatoglyph (**Figure 1B**). Surgeons can expose the thyroid gland through the gap between the sternal and clavicular heads of the SCM, or by dissecting the front edge of the sternocleidomastoid. Instead of opening the white line of the neck, this approach leverages the natural anatomical gap between neck muscles without severing the anterior cervical band to access the surgical field. Some reports (3, 4) showed that this approach had a smaller incision and could shorten hospital days due to the less postoperative drainage. By moving the incision to the lateral neck, which has less cutaneous tension, the postoperative scar is less remarkable and more easily concealed, contributing to better cosmetic outcomes (3–6). It also lowers the percentage of abnormal neck sensation and movement after surgery as the surgical field can be accessed by the natural anatomical gap between neck muscles (3, 5). The postoperative neck tissue damage reaction and scar adhesions are milder than those caused by COA. Apparently, this approach is suitable for patients without severe disease, especially for those who do not need total thyroidectomy and lateral cervical lymphadenectomy. This lateral approach can also considerably help surgeons with more feasible surgical fields when it comes to patients with a high BMI or a short neck.

Kim et al. (7) developed a new surgical approach, making a unilateral incision of 2.5–3.5 cm without crossing the midline along the skin crease in the lower neck (**Figure 1C**). In case of unilateral disease, the incision was made at the same side laterally, whereas for bilateral disease, in which case bilateral total thyroidectomy was performed, the incision was made on the side of the main lesion. The exposure of the gland increased by dissecting the plane between the SCM and the lateral edge of the strap muscle. The surgical procedures and advantages of this modified unilateral incision were similar to that of supraclavicular oblique incision. Because the incision in this approach is closer to the cervical midline, contralateral thyroidectomy is feasible without any restrictions; thus, total thyroidectomy is possible. These modified invasive open thyroidectomies described technically resemble the conventional open thyroidectomy, and each step in the procedure can be performed by the traditional approach that most surgeons are familiar with.

### 2.3 Minimally invasive video-assisted thyroidectomy

Paolo Miccoli preliminarily reported MIVAT in 1999 (8) before it was adopted worldwide thanks to its reproducibility and its comparable outcomes to COA. Although initially limited to benign thyroid nodules, MIVAT was progressively adopted for all types of thyroid diseases, while remaining within the selection criteria. The approach consists of a transcervical open technique through a 1.5- to 3-cm incision (**Figure 1D**) with the assistance of a 30°C 5-mm rigid endoscope and a video monitor. After the flap dissection, a special static suspension retractor is inserted into the incision to create the surgical space. The operation procedures are similar to the conventional open thyroidectomy.

The video assistance helps identify the structures and greatly minimize the incision and trauma. Thus, besides a better cosmetic result, it provides beneficial effects including decreased postoperative swallowing problems, better voice quality, less pain, faster recovery, and decreased incidence of wound-related complications (9). With regard to the safety of MIVAT compared with conventional thyroidectomy, pooled analysis of postoperative hypocalcemia and recurrent laryngeal nerve palsy have shown no significant difference (10). Lombardi et al. (11) reported that MIVAT has comparable oncologic results and the endoscopic view allows an accurate exploration of the central compartment and enables the identification of even slightly enlarged lymph nodes. Thanks to the uncomplicated procedures, MIVAT is easier and faster to learn compared with other endoscopic thyroidectomies. Thyroid surgeons can accumulate experience in endoscopic thyroidectomy through MIVAT. Although the operation time can be longer in the first several cases, some studies demonstrated that there was no statistical difference between MIVAT and COA (9). The mean operation time was 44.1 min (ranging from 30 to 130 min) for total thyroidectomy and 31.1 min (ranging from 20 to 120 min) for hemithyroidectomy according to Miccoli et al. (12), which are much shorter than other endoscopic thyroidectomies. Every coin has two sides; the shorter incision leads to a narrower surgical space, which makes it unsuitable for large thyroid volume or nodules. The endoscope may be unstable during the operation because there is no fixed support or much space for it. The most common complication is superficial thermal skin injury, although not commonly reported as a complication of traditional open thyroidectomy, which can occur with MIVAT and appears to be likely increased with smaller incisions (13).

MIVAT has opened new doors for endoscopic thyroidectomy. The emergence of MIVAT prompted thyroid surgeons to pay more attention to the improvement of cosmetic results. In the recent two decades, many different types of endoscopic approaches have been developed and some of them have achieved better cosmetic outcomes and have been widely adopted. Hence, MIVAT is not used as frequently as before since it still leaves a small scar on the anterior neck of patients.

Recently, this minimally invasive video-assisted strategy is used more frequently for functional lateral neck dissection (LND). Minimally invasive video-assisted lateral neck dissection (VALND) can greatly reduce the incision size, which used to require an extended collar incision or an L-shaped or U-shaped incision (sometimes approximately 20–30 cm), providing adequate exposure of the surgical field. After accomplishing thyroidectomy and central neck clearance, dissection was performed under endoscopic vision and instruments by using a technique very similar to conventional surgery through the single skin incision used for thyroidectomy. Several studies show that the safety and oncological completeness of VALND were similar to that of open procedures, but the VALND resulted in improved cosmetic results (14). Although the operation time is longer than conventional open LND, the video assistance provides a better exposure of level II LND, which can also be difficult for open LND. The VALND procedure is similar to the open procedure, making it less technically demanding and time-consuming.

## 3 Remote-access approach

### 3.1 Chest–breast approach

**Figure 2:**
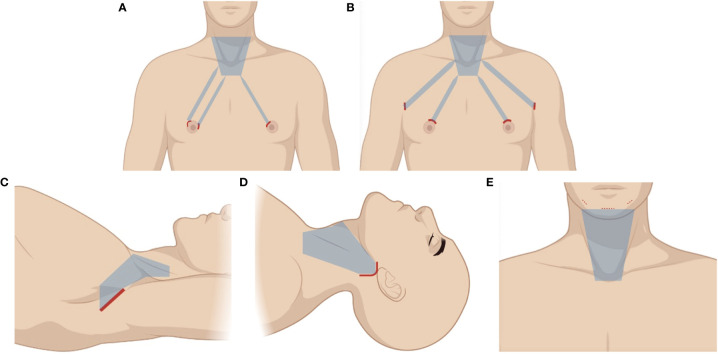
Illustration of different remote-access approaches. The area of raised skin flap was marked with blue wireframes. **(A)** Chest–breast approach (CBA). **(B)** Bilateral axillo-breast approach (BABA). **(C)** Transaxillary approach (TA). **(D)** Retroauricular approach (RA). **(E)** Transoral approach (TOA).

The chest–breast approach (CBA) was developed by Ohgami et al. in 2000 (15). Now, there are various types of CBA including approaches *via* the parasternum and bilateral mammary areolas (chest–breast) and total mammary areolas. As the technology advances, the total mammary areolas have drawn more attention, thus achieving the best cosmetic results among different types of CBA. One circumareolar 10-mm incision is made at the medial margin of the right areola for the endoscope. Then, two circumareolar incisions (approximately 5 mm) are made on the upper edge of the areolas on both sides (**Figure 2A**). After an injection of a diluted adrenaline solution into the subcutaneous space, subcutaneous and subplatysmal dissections are performed. After subcutaneous dissection, thyroidectomy is performed by using various endoscopic instruments. Total thyroidectomy can be accomplished despite not having a neck incision, which has excellent cosmetic outcomes, but it might leave a few small scars on the breast or chest to some degree, which is still unacceptable for some people, especially for women who care about the appearance of their breasts. There are some controversies when it comes to central neck dissection (CND). Because the surgical field is observed from the bottom during the operation, the sternum and clavicle are prone to block lower lymph nodes during CND. By lifting up the thyroid gland, the surgeons can leverage the traction of it to pull the central lymphatic tissue upward to excise them as a whole. Some studies indicate that this approach achieves equivalent therapeutic results to open surgery and conforms to the principle of radical tumor treatment for selected cases by well-trained surgeons. Zhang et al. (14) evaluated the effectiveness of CBA and COA regarding safety, cosmetic effects, and feasibility. There were no significant differences between the CBA and COA groups in postoperative calcium levels, parathyroid hormone levels, the total number of lymph nodes resected, and the number of metastatic central lymph nodes resected. Breast approach endoscopic thyroidectomy with LND has been previously described. Yan et al. (16) reported 10 years’ experience with the breast approach to patients with endoscopic thyroidectomy with level II, III, and IV LND. The retrieved lymph node number, complication rates, postoperative parathyroid hormone, and mean postoperative hospital stay were similar between the CBA and open group. The mean operating time in the CBA group (278.2 ± 38.6 min) was longer than that in the open group (179.3 ± 25.4 min). The recurrent rate was not significantly different (2/155 and 2/106) in the CBA and conventional open group. There is no doubt that this endoscopic LND requires excellent technique expertise. However, this approach needs a large amount of subcutaneous dissection and CO_2_ insufflation, which can diffuse into other tissue planes and even cause serious complications.

### 3.2 Bilateral axillo-breast approach

The bilateral axillo-breast approach (BABA), developed by Choe et al. (17) in 2007 as a modification of the unilateral axillo-breast technique described by Shimazu et al. (18), involves four ports placed around both areolae and axillae. Two 10-mm ports are placed over bilateral supra-areolar margins, 12–3 o’clock on the right and 9–12 o’clock on the left, serving as the camera and main working port and CO_2_ insufflation started. Another 10-mm port is placed at the axillary skin fold on the side of the operating surgeon, which serves as second working port. Specimen is retrieved through this port. Finally, a 5-mm port is placed at the opposite axillary skin fold (**Figure 2B**). A blind subcutaneous tunnel is created initially using a straight medium-sized hemostat forceps along the tangential lines directed towards the neck. After this, a large hemostat is inserted along the same tract followed by a metallic tunneler. Using to-and-fro motion, a plane is created over the chest and neck. A similar procedure is repeated on the opposite side. This is a blind procedure and one should be careful not to go too deep into the breast parenchyma or too superficial into the dermis, which causes bruising. This approach provides a symmetric anatomic view to the operators and allows the surgeon to have optimal visualization of crucial structures and to perform precise procedures in a wide surgical working space, resulting in improved clinical outcomes. The total thyroidectomy can also be performed through it. It is the only approach that allows four laparoscopic instruments to be used at the same time, which provides great convenience during surgery. Thus, it is the most common approach for robotic thyroidectomy. The operation time for lobectomy and total thyroidectomy is approximately 177.0 ± 40.8 min and 214.3 ± 40.8 min respectively, which are much longer than COA (19). The four long distant subcutaneous tunnels from the incisions to the neck require extensive flap dissection, and the use of CO_2_ insufflation is necessary. The complex procedure requires significant endoscopic surgical skills and the learning curve is steep. Alramadhan et al. (20) compared the surgical outcomes of BABA to COA in patients who had thyroid nodules with benign or intermediate fine-needle aspiration cytology results. BABA is comparable to COA in terms of complications and is safe and feasible when performed by experienced surgeons and for carefully selected patients who are concerned about neck scarring. However, BABA was found to be significantly associated with longer operation time, greater drainage volume, longer postoperative hospital stay, and higher average total medical expense. The CND is still controversial through this approach like CBA. Some studies compared the number of lymph nodes retrieved during CND in BABA and COA and reported a comparable number between the two procedures (21–25). However, in two propensity score-matched comparisons, the number of central lymph nodes retrieved was consistently lower in BABA than in COA (26, 27).

### 3.3 Transaxillary approach

The original transaxillary approach (TA) described by Ikeda et al. was performed with CO_2_ insufflation (28), which was then modified by Chung et al. in 2006 by utilizing a gasless approach (29). This approach involves a 5- to 6-cm incision in the axilla (**Figure 2C**), followed by the creation of a subcutaneous flap extending to the clavicle. By identifying the sternal and clavicular heads of the SCM, the central neck is entered, and a static elevating retractor is placed to maintain the surgical space; hence, CO_2_ insufflation is unnecessary. The procedure is done using a 30°C down camera and two endoscopic instruments. This approach also has excellent cosmesis because the incision is moved to the axilla, where it is hardly visible, and its surgical and oncologic outcomes are good (30). Jantharapattana et al. (31) reported a randomized study to compare the TA and COA. It showed that complications, such as vocal cord paresis and seroma, were not significantly different between the two groups. It also has remarkable advantage in identifying the recurrent laryngeal nerve and parathyroid glands, as well as in dissecting the upper pole or Berry’s ligament, because the surgical view and instrument direction are from lateral to medial. Additionally, compared with other types of endoscopic thyroidectomy, it provides a relatively larger surgical space, which makes it easier to operate on, particularly for large nodules or thyroid lobes. There might be some concerns about whether a greater area of dissected tissue and the prolonged retention time of the retractor can cause more pain. A meta-analysis demonstrated that postoperative pain was significantly lower on day 1 and day 7 in the endoscopic thyroidectomy group compared with the open thyroidectomy group (32). Kang et al. showed that there was no difference in postoperative pain between TA and COA (33), and because it approaches the thyroid through the posterior of the strap muscle and does not require a subplatysma muscle flap, the swallowing disorder and other abnormal sensations following thyroidectomy can be avoided or alleviated (34). TA has a longer operation time than COA, but less than other endoscopic approaches, because of its easier subcutaneous dissection and larger working space. Thus, we believe that it is an easier technique to grasp for surgeons without much endoscopic experience. A study of the learning curve for TA showed that the learning curve duration is approximately 60 cases for unilateral lobectomy. Thereafter, the learning curve of endoscopic total thyroidectomy is 38 cases (35). These advantages have made it become widely accepted by surgeons and patients and popular worldwide.

However, the biggest limitation of this technique is that it is not very convenient to remove the contralateral lobe with a unilateral incision compared with COA and other remote-access approaches such as CBA, and it needs a high level of expertise. Kim (36) has reported that single-incision, gasless, endoscopic transaxillary total thyroidectomy is a feasible and oncologic safe surgery. A 45° rigid endoscope was used to help the surgeon see downward easily and reduce the blockage of the trachea when detached to the contralateral side of the thyroid gland. The operation time of TA was significantly longer than COA (93.9 − 1.3 versus 142.6 − 3.3 min, *p* < 0.001). Total thyroidectomy through bilateral axillary approaches may be a choice for some special cases. It has to be admitted that the complexity of the procedure and the extensive subcutaneous dissection may be a huge challenge for the bilateral axillary approaches.

Ipsilateral CND is not difficult for TA with an en bloc resection, but the clavicle may be a shelter for deep regions. Kim et al. reported a significantly lower number of lymph nodes being harvested in the TA group than in the COA group, while the number of positive lymph nodes between the two groups did not differ significantly (30). A robotic assistance can be used in TA to improve manual dexterity, ergonomics, and visualization and help remove contralateral thyroid lobes. LND can be performed for strictly selected patients, especially with the robotic assistance, but it has several technical difficulties and limitations including the following: (1) a longer operation time; (2) a wider flap dissection is needed, which means greater trauma and more severe paresthesia of the flap area; (3) higher costs; (4) a prominent clavicle may make it hard to completely remove lymph nodes of level IV; (5) the upper neck (level II) is difficult to remove; and (6) splitting the SCM may exaggerate neck pain and stiffness, especially with bilateral procedures (37), which make it difficult to routinely adopt.

To shorten the flap distance, some surgeons try to modify the transaxillary approach by moving the incision under the clavicle along the dermatoglyph. A modified static retractor is used to retract the flap, the sternal head of the SCM, and strap muscles. The surgical procedures and techniques are the same as transaxillary endoscopic thyroidectomy. Despite the cosmetic result being not as good as the transaxillary approach, it still grants the request of some patients of not leaving a scar on the neck. Because the incision is close to the clavicle and thyroid, the visual field and operative space are more flexible. The minimal surgical trauma is another advantage of this approach compared with other endoscopic thyroidectomies.

### 3.4 Retroauricular approach

The retroauricular approach (RA) described by Terris et al. (38) entails an L-shaped incision behind the auricle, and a flap is created above the SCM, proceeding anteriorly to the midline of the neck and extending superiorly to the submandibular gland and inferiorly to the level of the clavicle and sternal notch (**Figure 2D**). The flap is subsequently retracted and maintained by a self-retaining retractor. The anterior border of the SCM is delineated and retracted posteriorly to reveal the carotid sheath located lateral to the ipsilateral lobe of the thyroid gland. The omohyoid muscle is identified and skeletonized. After retracting the SCM muscle posteriorly and the omohyoid muscle superiorly, the exposed strap muscles are also dissected at the lateral side and maintained superiorly by the retractor to reveal the superior pole of the thyroid gland. Then, the contour of the thyroid gland is fully exposed and a sufficient working space is established with the self-retaining retractor, instead of CO_2_ insufflation. The followed procedures are similar to those of TA. The flap distance in RA is the shortest of all the remote-access techniques; thus, a smaller area of dissection compared with other endoscopic approaches will result in less tissue trauma. However, access to the contralateral lobe is limited. The inevitable cervical sensory nerve damage during flap elevation causes pain or dysesthesia above the involved neck area. Moreover, the incision scar can sometimes be disfiguring due to the long-term retraction damage to the incisional edge along the posterior hairline (39). Because the RA and TA have much in common, several studies have compared these two techniques. The RA approach seems to be beneficial for reducing operation time and hospital stay, and for stabilization of the learning curve (40). The view from the top down makes it easier to address level VI lymph nodes compared with the TA, while the TA provides a larger surgical space and more familiar vision of anatomical structure. LND *via* RA is technically feasible and seems safe with satisfactory cosmetic results for patients (41). Because the incision is much closer to the lateral neck area, the exposure of lateral cervical lymph nodes is more adequate and the dissection can be performed more conveniently, but only for the ipsilateral neck.

### 3.5 Transoral approach

The most popular type of transoral approach (TOA) is the transoral endoscopic thyroidectomy *via* vestibular approach (TOETVA), which was first described by Richmon et al. in 2010 in a preclinical study using cadavers. Then, the TOETVA using laparoscopic instruments was popularized in 2015 after a study by Anuwong describing 60 cases of successfully performed procedures in Thailand was published (42). The patient is placed in the supine position, with slight neck extension, which is important to the subcutaneous dissection, because the mandible might be a block. Three laparoscopic ports are inserted under the lower lip at the oral vestibular area. The first incision is made transversely and centrally at two-thirds of the distance between the inferior labial frenulum and the edge of the lower lip. The length of the first incision can vary between 1.5 and 2.5 cm depending on the size of the thyroid gland. A 10-mm trocar is inserted through the first incision as a camera port. The hanging stitch is made at 1 cm below the tip of the trocar to prevent an acute angle of the skin flap in front of the port. Two of 5-mm working trocars were inserted at both lateral sides of the oral vestibule. The working space is created down to the sternal notch with the lateral border at the SCM (**Figure 2E**). The strap muscles are separated in the midline and retracted laterally by a transcutaneous 2/0 silk suture to expose the thyroid and trachea. Obviously, the biggest advantage of TOETVA is that it is the only approach that does not leave a scar. Total thyroidectomy is not limited anymore in TOETVA. With an up-to-down view, the exposure of central lymph nodes is more effective without the blockage of the sternum and clavicle. It also has a closer access to the anterior neck structures and the thyroid than other remote-access approaches, which can decrease the trauma of extensive subcutaneous dissection. Many studies have testified that TOETVA can be performed safely and has outcome and complication rates similar to those of open thyroidectomy (43). Some reports even show decreased postoperative pain and swallowing difficulties, better voice quality, faster recovery, and decreased incidence of wound-related complications (44, 45). However, it also has some drawbacks. Given the restricted area of oral vestibule, the three inserted trocars are very close to each other, leading to the limitation of the movement of the endoscopic equipment. In addition to the narrow surgical field of the neck, it requires a skilled surgeon to perform the surgery. Previous studies demonstrated that 11–40 cases were required to achieve technique proficiency of transoral thyroidectomy (46–48). The instruments used in the TOA were inserted through working ports in the oral vestibule and down toward the neck, in a cranial-to-caudal direction. If the thyroid tumor is located in a high-riding upper pole, this direction of the instruments would make it very difficult to remove it adequately and completely (49). The mental nerve might be injured during the incision, characterized as lower lip paresthesia after surgery. The CO_2_ insufflation is usually needed, and along with the limited suction and irrigation capabilities within the narrow neck air pocket, the surgical field may become obscured by smoke and blood such that the assistant frequently needs to clean the endoscope (50). The operation time is significantly longer compared with open thyroidectomy (51). The resected specimen extract from the middle incision needs to be broken into pieces to avoid mental nerve injury when the specimen is too large to be removed as a whole, a situation that may result in deviation from the standard oncological surgery for malignant nodules. Wu et al. suggested that the safest malignant nodular diameter is 20 mm, with 100% sensitivity and 87.5% specificity (52). Infection is another concern, because transoral incision is categorized as a clean contaminated wound. However, few cases of infection have been reported in a series of studies about TOETVA (43, 53, 54). Whether antibiotics should be used during the perioperative period to avoid infection needs further study. Recently, some improvement measures have been attempted on TOETVA. For example, some special instruments and suspension systems for gasless TOETVA have been developed to avoid the complications of CO_2_ insufflation (55).

Transoral endoscopic thyroidectomy *via* the submental approach is a modification of TOETVA. The middle incision on the oral vestibule is replaced by the incision on the natural skin depression immediately under the chin. The submental approach can reduce the difficulty of the placement of the 10-mm central trocar requiring the detachment of the chin tissue from the mandibular periosteum. It also has many other advantages compared with TOETVA such as it decreases the lower lip trauma and, consequently, potential lesions of the mental nerves (medial branches) with a lower incidence of postoperative dysesthesia; it eliminates the conflict between trocars thanks to better instrument triangulation (56). Thus, it might be an easier option for “invisible scar” thyroidectomy.

## 4 Selection strategy

Above all, we can draw the conclusion that each approach is imperfect and has its own advantages and disadvantages. Referring to the reported studies and our own experience, we summarized the performance of the different approaches in **Table 1**. It is just for reference, as there must be controversies in some aspects among surgeons, and further studies are needed. There is no doubt that the remote-access approaches can provide better cosmetic outcomes compared with cervical approaches since there is no visible scar on the neck. TOA has the best cosmetic outcome among all approaches as it does not leave a scar. Because the incision is made on the side of the body, it may not be very convenient to remove the contralateral thyroid gland or lymph node through LA, RA or TA, especially for beginners. As shown in **Figures 1**, **2**, the size of the raised skin flap in remote-access approaches is much bigger than that in cervical approaches, which could mean more operative trauma. The remote-access approaches require more operation time and higher skills given the complex surgical space and the use of endoscopic instruments. Lee has compared the operation time of hemithyroidectomy with or without central lymph node dissection through different remote-access approaches. The research showed that TOA had the longest operation time (57). It is obvious that, except for MIVAT, cervical approaches can provide more adequate working space, especially COA. Given the large amount of dissected tissue and the use of a wide static elevated retractor, TA can provide a larger working space compared with other types of endoscopic thyroidectomy. TOA and RA have an advantage over CND with an up-to-down view, while CBA, BABA, and TA are the opposite, because the blockage of sternum or clavicle may be concerning. MIVAT can help to identify lymph nodes in the deeper area of central compartment. Although endoscopic LND is a feasible and safe technique in terms of complete resection of the selected neck levels (37), COA is still the first choice for LND.

**Table 1:** Comparison of different approaches’ performance in different aspects.

Approach	Cosmesis	Bilateral operation	Operative trauma	Operative time	Operative complexity	Learning curve	Working space	CND	LND
COA	☆	★★★★★	★★	★	☆	☆	★★★★★	★★★★	★★★★★
LA	★★	☆	★	☆	★	★	★★★☆	★★★	–
MIVAT	★★	★★★★	★	★★	★☆	★★	☆	★★★★★	★★★★
CBA	★★★★	★★★★	★★★★	★★★★	★★★★	★★★★	★★	★★	★★★
BABA	★★★☆	★★★★	★★★★★	★★★★☆	★★★★	★★★★☆	★★	★★	★★★
TA	★★★☆	★	★★★★	★★★	★★	★★★	★★★	★★	★★
RA	★★★☆	★	★★☆	★★★	★★★	★★☆	★★	★★★★	★★★
TOA	★★★★★	★★★★	★★★	★★★★★	★★★★★	★★★★★	★	★★★★★	☆

The extent is measured by the number of stars. “☆” represents a half of “★”. “-”means inapplicable.

Since the patients do not have much knowledge of these novel technologies and most of them will take the doctor’s advice, it is very important to select the most appropriate way based on different situations in case of the abuse of these new techniques, which can lead to poor treatment outcomes or increased unnecessary burden on patients. As surgeons, there is no doubt that we must first ensure treatment success and the safety of the surgery. Moreover, even if there are no universal selection criteria for these new techniques, the following elements need to be considered before surgery.

### 4.1 Patients’ desire for cosmesis

Being attractive is innate to humans, but people value it differently. The influencing factors include gender, age, occupation, financial condition, personality, and culture. All cosmetic surgery should be based on the patients’ desire for beauty. When making the surgical plan, we must respect the patients’ opinions, without violating the therapeutic principle, rather than imposing our will on them. Patients with strong requirement for cosmesis would prefer remote-access approaches, which have a better cosmetic outcome, even though they are usually associated with more trauma, longer operation time, and greater costs. TOETVA has the best cosmetic results (57), especially for patients with scar diathesis, among all the approaches as it leaves no scars. In contrast, cosmetic thyroid surgery is not necessary if patients do not wish for a scarless approach. In this case, LA may be a choice owing to its advantage in alleviating postoperative cervical discomfort, but more studies are needed to confirm its outcomes.

### 4.2 Bilateral operation

As mentioned above, bilateral operation can be very difficult or infeasible for some approaches such as LA, RA, and TA. To perform bilateral operation, COA has advantages in almost all aspects except for cosmetic outcome. The operation time, difficulty, and trauma of COA are much less than any other approaches. For the patients who need to undergo total thyroidectomy but have cosmetic requirements, TOA may be their first choice for now. Nevertheless, some people may be terrified of surgery and reject this approach; thus, CBA and BABA should be considered. TA and RA can meet the cosmetic requirements of most patients who just need a unilateral operation, and the shorter operation time and lower difficulty make them a better option in most situations.

### 4.3 Size of the nodule and thyroid

It is recommended that the nodule should not be more than 3 cm and the thyroid should not be less than 5–6 cm in the largest dimension for these novel approaches (2). The enlarged gland usually has an abundant blood supply and a smaller working space, which increases the risk of intraoperative bleeding. Yet, the recommended size of the nodule and thyroid can be different from different approaches and surgeons. The limitation can be extended to some degree since TA can provide a larger working space, while we should be cautious about it for TOA. It depends on the experience and proficiency of the surgeons as well.

### 4.4 Location of the lesion

Substernal goiter can be a challenging disease, and the thyroid surgeon should approach it with respect. The gland can extend to critical structures such as great vessels and pleura. Given the high surgical risk, careful operation and adequate exposure are required to avoid disaster, which means COA is often the only option. Sometimes, sternotomy is mandatory. If the malignant lesion is adjacent to the entrance point of the recurrent laryngeal nerve, the remote-access approach should be used with caution. The use of an energy-based device in endoscopic surgery is essential, which needs a safe distance when cutting the tissue to avoid thermal damage to the recurrent laryngeal nerve. It is difficult to guarantee the complete removal of the tumor and avert nerve injury when the tumor is too close to the entrance point. Moreover, the lesion in the upper pole of the gland can be difficult to dissect *via* TOA.

### 4.5 Peripheral tissue invasion

If the preoperative examinations show evidence of thyroid cancer with extrathyroidal extension, such as laryngeal nerve and trachea, which is always an absolute contraindication for these novel approaches, COA is the best choice.

### 4.6 Central lymph node metastasis

Central lymph node metastasis is very common in thyroid cancer, found in 32.4% to 84.3% of clinically node-negative papillary thyroid carcinoma patients who underwent total thyroidectomy and bilateral central lymph node dissection (58). However, the ability of preoperative ultrasonography to identify central lymph node metastasis is limited due to the overlying thyroid gland, which means only obvious metastasis can be found preoperatively. TOA has advantage on CND over others given the superior view. A propensity score-matching analysis showed that there were no significant differences in the mean number of central lymph nodes retrieved between the TOA group and the COA group (9.39 ± 4.01 versus 10.71 ± 5.17, *p* = 0.202) (59). Sun (60) reported that the number of dissected lymph nodes (7.0 ± 5.3 versus 7.3 ± 3.9, *p* = 0.681) and the number of metastatic lymph nodes (2.0 ± 2.5 versus 1.8 ± 1.7, *p* = 0.723) did not differ significantly between COA and CBA groups. He’s research (25) showed that there was no significant difference in the number of central lymph node removed between COA and BABA (6.7 ± 2.0 versus 6.8 ± 2.1, *p* > 0.05). Kim et al. reported a significantly lower number of lymph nodes being harvested in the TA group than in the COA group (5.5 ± 4.2 *vs*. 8.3 ± 6.2, *p* < 0.001), while the number of positive lymph nodes between the two groups did not differ significantly (30). In general, many studies have reported that CND through remote-access approach had equivalent outcomes to COA, but the subjects included in almost all studies are strictly selected. Hence, if central lymph node metastasis is indicated by the preoperative examinations, much more attention should be paid to approach selection.

### 4.7 Lateral lymph node metastasis

Lateral lymph node metastasis, not rare in patients with thyroid cancer, is related to worse prognosis and higher recurrence rate. To perform LND, a complicated operation in itself, an extended incision is often created, causing unsatisfactory experiences after the surgery. Although several studies about endoscopic LND have been reported in recent years, there is still a lot of controversy about it. Data are not sufficient to draw conclusions about oncologic equivalency. The completeness of the surgical resection in selected compartments is satisfactory; however, some neck levels are difficult to achieve. So far, it is not a routine treatment, and only strictly selected patients are eligible for this technique. If the metastatic lymph nodes are fused or fixed, or the largest lymph node diameter is >2 cm, COA should be first recommended whether for CND or LND. VALND is not a bad choice, which is characterized by much less trauma, close to open vision, and being not limited by local tumor progression, and it can also provide a clearer vision of some covert areas.

Other factors should be taken into account such as history of neck surgery, BMI, occupation, comorbidities, histological type, and surgeon’s proficiency.

### 4.8 Summary

**Figure 3:**
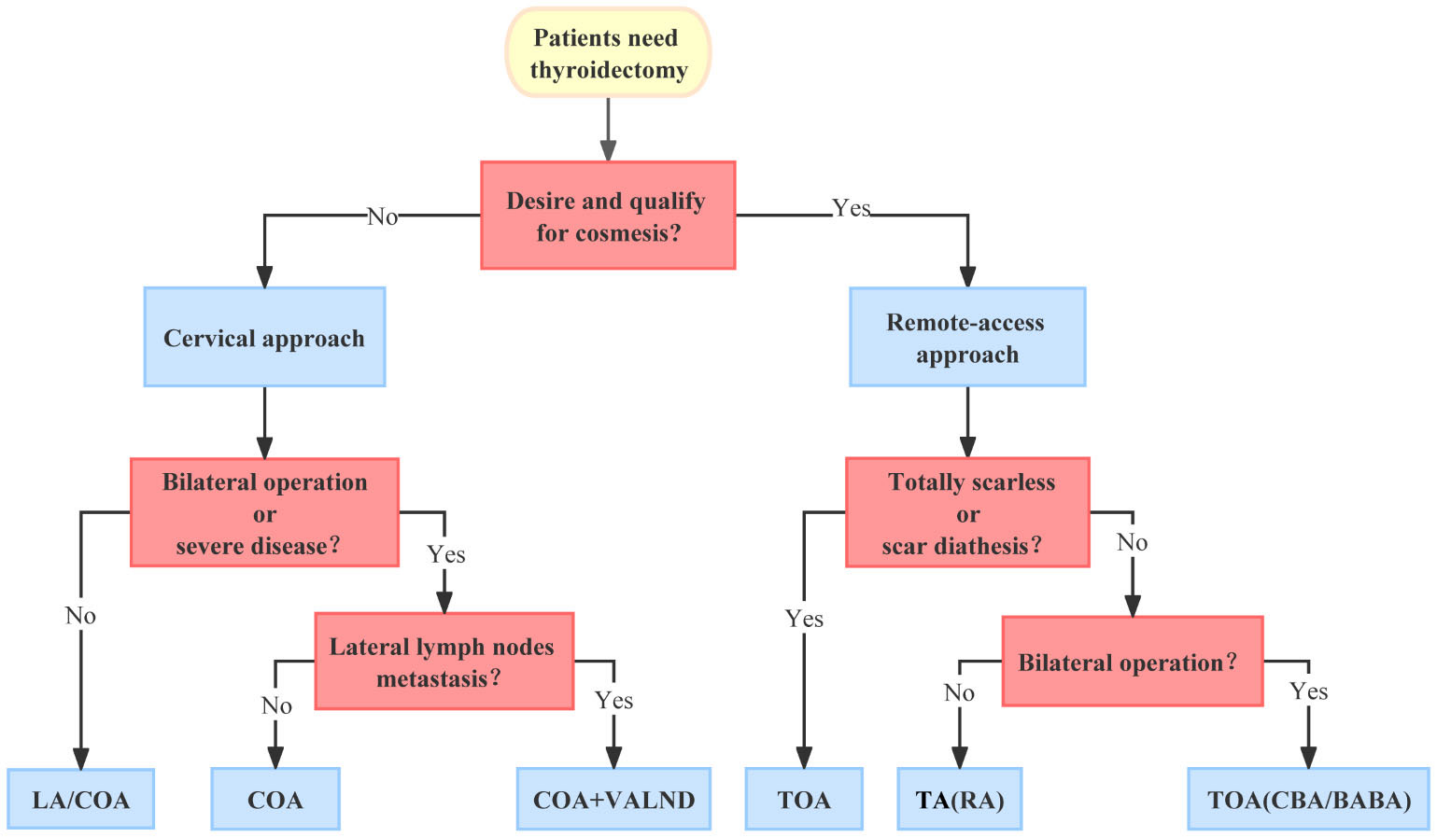
The flowchart of the approach selection.

As presented in **Figure 3**, we tried to summarize an approach selection strategy as reference for ordinary situations since it is impossible to cover all cases and individualized therapy should be advocated. First of all, we should decide whether a cervical approach or a remote-access approach is needed. The ideal criteria of remote-access approach include the following (1): patient’s demand for cosmetic outcome; (2) well-circumscribed nodule ≤3 cm and thyroid lobe <5–6 cm in the largest dimension; and (3) underlying thyroid pathology with no evidence of thyroiditis on ultrasound. Exclusion criteria are as follows: (1) evidence of thyroid cancer with extrathyroidal extension or lymph node involvement; (2) Graves’ disease; (3) substernal extension; and (4) previous neck surgery (2). If the remote-access approach is chosen, the next procedure is to decide the specific approach. For patients with scar diathesis, those who wish to have no scars, or those who need to undergo bilateral total thyroidectomy, TOA might be the best option. CBA and BABA can be the backup of TOA for isolated instances. For patients who just need a unilateral operation, we suggest TA should be considered first because of its simpler procedure and larger working space; RA is also not a bad option. If the remote-approach is unnecessary or inapplicable, the cervical approach should be adopted. LA can be suggested to patients with mild disease, who just need unilateral lobe dissection. For the rest of the patients, COA should be the main approach. The video-assisted approach is mainly recommended to patients who need LND to shorten the incision and alleviate surgical trauma.

## 5 Conclusions

The technological progress and idea changes have brought about many alternative surgical techniques to the thyroid gland, and some of them have been widely used to improve cosmetic and functional outcomes. Meanwhile, these novel techniques may result in new problems if not used appropriately. To achieve individualized treatment, we need a comprehensive understanding of the characteristics of various approaches and incisions. COA is still the main approach, which plays an irreplaceable role in thyroid surgery. TOA and TA have drawn more attention in clinical application due to their characteristics in recent years. MIVAT and other approaches also have their own unique advantages. These new techniques can complement each other to solve the problems of different patients. However, further studies are needed to evaluate the long-term outcomes of these new techniques. We believe that the continuous development of new techniques and instruments will help patients in terms of better therapeutic and cosmetic efficacy.

## Author contributions

QL, XZ, and GC contributed to the conception and design of the study. SX and PW supervised the paper. QL wrote the first draft of the manuscript. All authors contributed to manuscript revision, and read and approved the submitted version.
